# (Hydroxypropyl)methyl Cellulose-Chitosan Film as a Matrix for Lipase Immobilization—Part ΙΙ: Structural Studies

**DOI:** 10.3390/gels8090595

**Published:** 2022-09-17

**Authors:** Evdokia Vassiliadi, Marta Tsirigotis-Maniecka, Henry E. Symons, Pierangelo Gobbo, Frédéric Nallet, Aristotelis Xenakis, Maria Zoumpanioti

**Affiliations:** 1 Institute of Chemical Biology, National Hellenic Research Foundation (NHRF), 48 Vassileos Constantinou Ave., 11635 Athens, Greece; 2 Department of Biological Applications and Technology, University of Ioannina, 45110 Ioannina, Greece; 3 Faculty of Chemistry, Wrocław University of Science and Technology, 50-370 Wroclaw, Poland; 4 School of Chemistry, University of Bristol, Bristol BS8 1TS, UK; 5 Department of Chemical and Pharmaceutical Sciences, University of Trieste, 34127 Trieste, Italy; 6 Centre de Recherche Paul-Pascal, University Bordeaux, CNRS, UMR 5031, 115 Avenue du Docteur-Schweitzer, 33600 Pessac, France

**Keywords:** hydroxypropylmethyl cellulose, characterization, blended polymers, film, enzyme immobilization, lipase

## Abstract

The present work reports on the structural study of a film made of a hybrid blend of biopolymers used as an enzyme carrier. A cellulose derivative (HPMC) and chitosan (CS) were combined in order to formulate a film on which *Mucor miehei* lipase was immobilized. The film was successfully used as a biocatalyst; however, little is known about the structure of the system. Therefore, small-angle X-ray scattering, Fourier transform infrared spectroscopy (FTIR), optical microscopy, and scanning electron microscopy (SEM), as well as microindentation measurements, were used to shed light on the structure of the promising biocatalyst. Among the results, intermolecular hydrogen bonds were observed between the amide groups of the two polymers and the lipase. The presence of the enzyme does not seem to affect the mechanical properties of the matrix. The used film after 35 cycles of reaction seemed to be fatigued and had lost part of its humidity, explaining the reduction of the enzyme activity.

## 1. Introduction

Natural-polymer-based matrices are widely used for enzyme immobilization [1] due to their practical significance in the design of biocatalytic hydrogels/membranes/vessels for industrial synthesis [2,3]. In addition, compared to free enzymes, immobilized ones are more resistant to environmental changes. Furthermore, with enhanced stability, immobilized enzymes can be easily separated from the reaction mixture and reused, thereby simplifying the separation and recovery processes [4,5].

Enzyme immobilization can take place by physical or chemical methods [6]. Physical methods, where no covalent bonds appear, can be either absorption [7,8] or entrapment [4,9], where enzymes are adsorbed on the surfaces of support carriers or occluded in polymeric networks, respectively. In these cases, no additional coupling agents and modification steps are required for the immobilization, offering thus a low-cost preparation. The performance of the immobilized enzymes depends on the properties of the supporting material used as well as the composition and structure of the immobilization matrix.

Cellulose, chitin, and their derivatives are attractive materials for enzyme immobilization [10]. They are the two most abundant natural polymers. The matrices they produce do not alter under physiological conditions. In addition, their hydrophilic/hydrophobic character can be adjusted according to the needs of the application [11,12]. While in most cases, the formation of hydrogels that are based on native cellulose or chitin is a two-step process which involves firstly dissolution and afterwards cross-linking (i.e., gelation) [13], there is also a different approach by which only the physical interactions affect the linking of the two polymers. In the latter case, the derivatives of the polymers are most commonly used as they are much easier to fabricate into hydrogels because they are water- or acid-soluble [14]. To add to this, cellulose and cellulose derivatives are the most studied polymers as they are biocompatible, cost-effective, and renewable [15,16].

For film carriers, an understanding of the microstructure of the film is critical since it determines the advantages and disadvantages for their potential application. Several studies offer a detailed analysis of polymer mixtures morphologically and structurally [15,17,18]. The techniques used aim to reveal the structure and the film surface image, as well as to present the interaction between the system’s compounds [19,20].

The present study is an attempt to understand the morphology and structure of a well-studied biocompatible film, in an effort to provide insight into the performance of the biocatalyst that has been proven to give good results [21]. Having already studied the optimal conditions in which the film as a biocatalyst could perform on the model reaction of propyl laurate synthesis, the first morphological results were obtained with the use of atomic force microscopy. The results triggered the interest to perform a deeper analysis on this easily reusable biocatalyst [21]. Therefore, the aim of the present study is to structurally characterize a biocatalyst based on the blend of two polymers, namely, (hydroxypropyl)methyl cellulose (HPMC) and chitosan (CS). For this purpose, small-angle X-ray scattering (SAXS) and Fourier transform infrared spectroscopy (FTIR) studies were conducted, whereas morphological analysis was conducted via optical and scanning electron microscopy (SEM). For the physicochemical studies, the electrophoretic zeta potential (ζ) was measured, and the moisture content of the films was determined gravimetrically using a moisture analyzer. Lastly, to investigate the mechanical properties, microindentation measurements were carried out.

## 2. Results and Discussion

**Figure 1:**
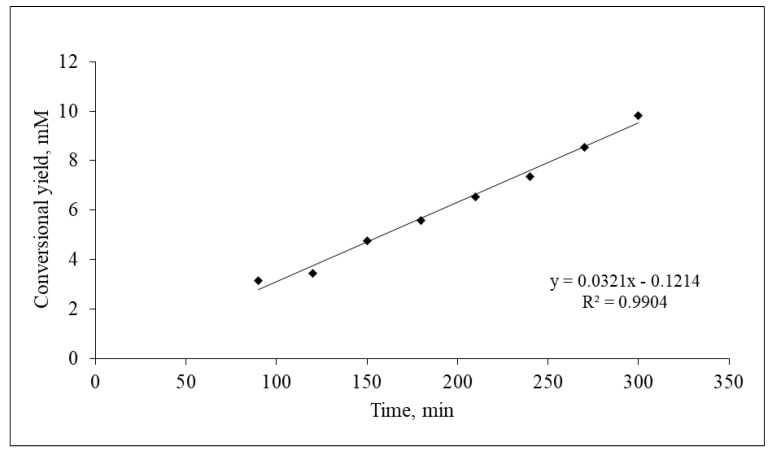
Reaction profile, for up to six hours, of the esterification of 1-propanol with lauric acid (100 mM, each) catalyzed by *M. miehei* lipase immobilized on HPMC:chitosan film (2:1 ratio). Lipase concentration, 0.3 mg/g of film; 10 mL of isooctane as solvent, at room temperature.

The purpose of the present study is to thoroughly describe the structure of a biocatalyst used successfully in a previous work [21]. In this work, the two polymers (namely, HPMC and chitosan) were combined in order to formulate a film on which *Mucor miehei* lipase was immobilized. To investigate the ability of the matrix to maintain the enzyme’s catalytic activity, the biocatalyst was tested towards the model esterification reaction of propyl laurate synthesis, a 6 h profile of which can be seen in Figure 1.

In that perspective, several parameters were studied. The ratio of the polymers used was examined and the ratio HPMC:CS = 2:1 proved to form the most promising matrix. Increasing the amount of the immobilized enzyme appears to improve the reaction yield, indicating, however, mass transfer limitations. The optimum catalytic performance was observed for the biocatalyst with enzyme:polymer ratio = 1/200. The enzyme-loaded film presented a remarkable reusability, since it could be used for up to 35 times without loss of activity [20].

Before small-scale investigation of its properties, several macroscopical observations were made. Each polymer solution mixture, regardless of the HPMC/CS ratio used to prepare it, was macroscopically homogeneous [22]. When only HPMC was used for film preparation, it resulted in a transparent, colorless film, whereas in the presence of chitosan, the films prepared had a slightly yellowish color. This is in accordance with previous reports on films that contained chitosan [17]. Furthermore, the presence of the immobilized enzyme provided an opaque effect on the film.

The aim of the study is to structurally characterize the biocatalyst which will be proposed for the synthesis of high-added-value products of industrial interest.

### 2.1. Structural Studies of the Biocatalyst via Spectroscopic Methods

#### 2.1.1. Small-Angle X-ray Scattering (SAXS)

**Figure 2:**
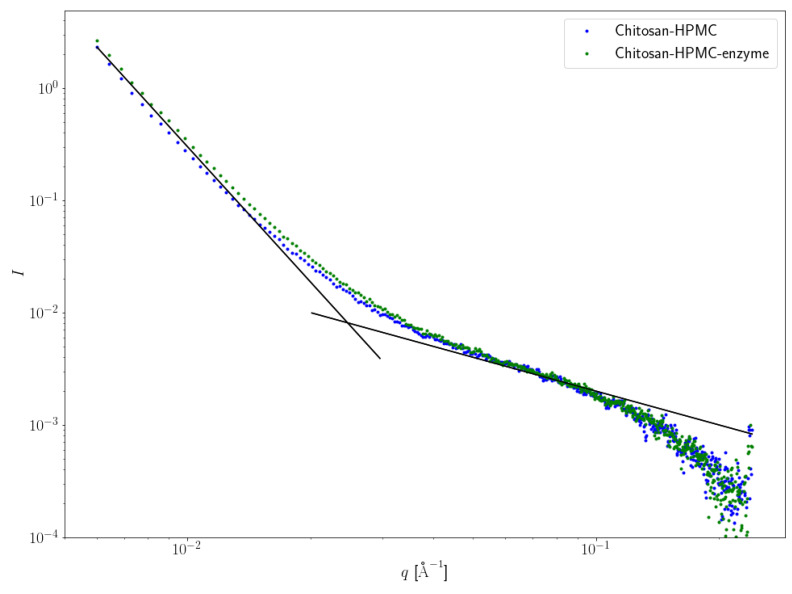
Processed (background-subtracted) data; double logarithmic scale; SAXS spectra for HPMC:CS films. Blue curve: empty film; green curve: enzyme-loaded film. HPMC:CS = 2:1. Enzyme: *M. miehei* lipase, 1 mg/g of film. The two superimposed black lines correspond to power-law decays, as 1/q⁴ and 1/q, respectively.

The structural studies of the HPMC–CS films were conducted using two spectroscopic techniques to reveal any alterations of the polysaccharide-based matrix after lipase incorporation. The first approach involved using a small-angle X-ray scattering (SAXS) technique that is an analytical and nondestructive method used to investigate nanostructures in liquids and solids. SAXS is able to probe the length scales of 10–1000 Å and therefore is an appropriate method to determine the size and the structure of gels and films. In the present study, this method was used in order to clarify the possible alterations that may be provoked in the film matrix after the incorporation of the enzyme. Figure 2 represents the intensity profile of the empty (blue curve) or loaded with enzyme (green curve) film.

According to Figure 1, adding the enzyme to the HPMC–CS system has very little structural effect at scales probed by SAXS. The intensity upturn observed for low q values (below *circa* 2.5 × 10^−2^ Å^−1^), with a scaling 1/q⁴, as commonly observed in locally heterogeneous systems such as gels, is less pronounced for the enzyme-free film. Supramolecular objects in the reference HPMC–CS, as well as for enzyme-loaded HPMC–CS films, look similar to elongated cylinders, as suggested by the (not fully developed) intermediate regime scaling such as 1/q with very similar diameters, since the large-q crossover from the intermediate regime to the asymptotic regime (also known as Porod’s law) occurs in the same scattering wave vector range, about 15 Å^−1^ [20].

#### 2.1.2. Fourier Transform Infrared (FTIR)

**Figure 3:**
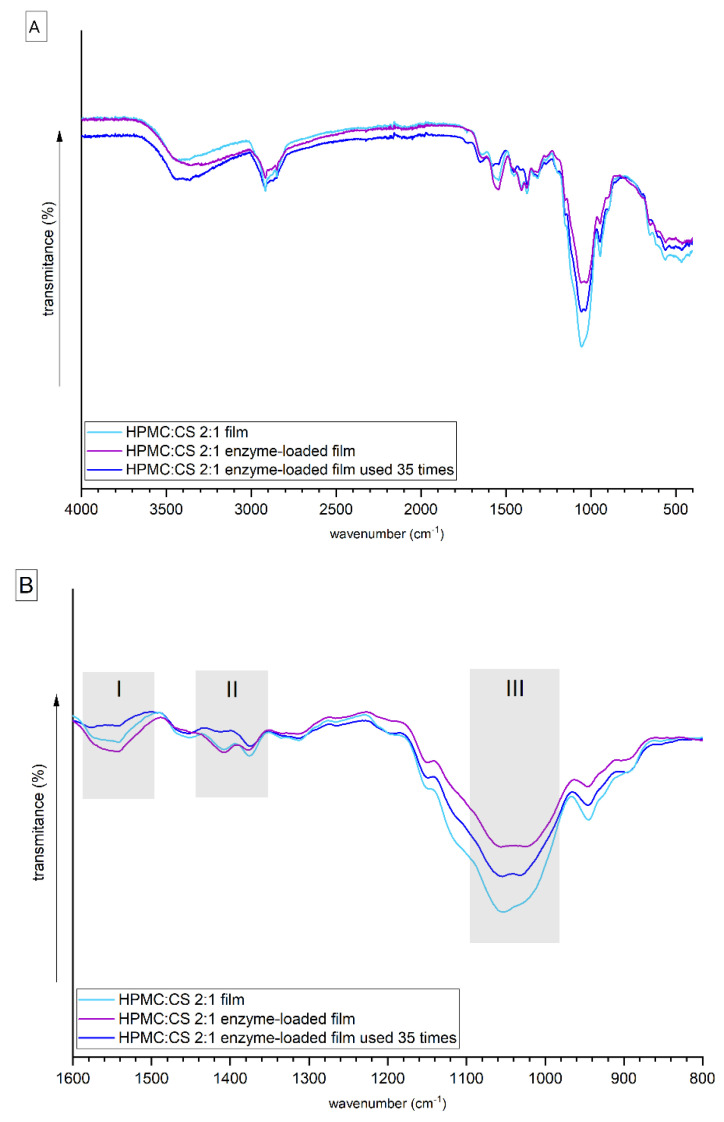
(**A**) Full FTIR spectra for HPMC–CS film. Light blue curve: unloaded film; purple curve: enzyme–loaded film; blue curve: enzyme–loaded film after 35–times usage cycles. (**B**) Focused FTIR spectra from 800 to 1600 cm^−1^. HPMC:CS = 2:1.

Fourier transform infrared (FTIR) spectroscopy is a nondestructive technique which can be used to monitor discrete changes related to the interactions between specific functional groups of the constituents of the HPMC–CS films. Several absorption bands typical for polysaccharides and lipase were observed in the spectra (Figure 3). The wide band observed in the 3600–3200 cm^−1^ corresponds to overlapped signals from inter- or intramolecular stretching vibrations of the OH and –NH groups [16], and symmetrical and asymmetrical vibrations of the NH_2_ groups [23]. The signals visible around the 2900 cm^−1^ shift are assigned to the stretching vibrations of the C–H bonds in the propyl group of HPMC. The band located around shift 1650 cm^−1^ (the most pronounced for the HPMC:CS = 2:1 used film) can be ascribed to amide I [24]. Signals visible in region I (~1545 cm^−1^) (Figure 3B), assigned to the amide II band, which is a combination of –NH_2_ in plane bending and C–N stretching vibrations, are the most pronounced for the lipase-loaded film. Additionally, the presence of these signals confirms the formation of intermolecular hydrogen bonds between the amide groups of CS and lipase and hydroxyl groups of HPMC [24]. The signals in region II (~1410 cm^−1^) (Figure 3B), assigned to the in-phase combination of the vibration of N–H deformation with C–N in amides III with a contribution of symmetric deformation from –COO^−^ [3,25], are visible for films with or without lipase. The signals visible in region III (Figure 3B) in the range of ~1060–1020 cm^−1^ can be ascribed to the stretching skeletal vibrations of the C–O–C bridge of the pyranose ring in HPMC, and asymmetric deformation of –COO^−^ [24] are the weakest for the unloaded film, while the signal from –COOH (1738 cm^−1^) is visible only in the spectrum of the film used. When comparing the spectroscopic behavior of the unloaded film and the enzyme-loaded film, it can be confirmed that there are some weak electrostatic interactions between the polysaccharides and the lipase.

### 2.2. Physicochemical Studies of the Biocatalyst

The electrostatic interaction between the enzyme and the polysaccharides is also confirmed by measurements of the zeta potential of aqueous solutions of polysaccharides and the enzyme. The ζ values of 2% solutions of HPMC and CS were as expected, typical for these polysaccharides [26], −1.94 ± 3.82 and 33.35 ± 4.53, respectively. The ζ value of polyelectrolytes solutions is the result of a surface charge that strongly depends on the pH of the environment due to the presence of ionizable functional groups within their structure, i.e., amine in CS and carboxylic in HPMC [27]. The mixing of HPMC and CS solution caused an increase in the pH of the solution from ~2.0 (for CS) to ~5.0 (for HPMC:CS = 2:1); thus, the amine groups were still partially protonated, while carboxylic groups were already partially deprotonated. The attractive electrostatic interactions between COO and NH_3_^+^ (2:1) caused a significant neutralization of the surface charge of polyelectrolytes, which greatly reduced the number of positive charges and affected the zeta potential value to ζ = 18.95 ± 6.22. Adding the enzyme to the HPMC–CS mixture caused an increase of ζ value to 21.90 ± 5.10, which may suggest that the amine groups present in lipase also exploit carboxylic groups of HPMC, further neutralizing the surface charge.

The aqueous environment is beneficial for enzymes, including lipases, and thus enzyme immobilization in hydrophilic polymeric structures, i.e., matrices, helps to maintain their catalytic activity. Although the fabricated biocatalyst is designed to work in organic media, the presence of residual water molecules may be desirable for the enzyme and may be important to keep the polysaccharide-based matrix physically intact. The dehydration of the hydrophilic matrix may lead to weakening of its structure and reduced resistance to mechanical disturbance. Loading the films with enzyme caused more water molecules to remain trapped within the matrix, as the unloaded film had 1.4% moisture content, while the moisture content increased for the loaded film to 2.7%. Water molecules trapped in the film structure provide the conditions for the enzyme to remain active, but when the moisture content was reduced to 0.9% for the HPMC–CS enzyme-loaded film after 35 uses, not only did the enzyme activity drop significantly [21], but those nonpolar and unfavorable for polysaccharides conditions also influenced the morphology of the film. Thus, the results suggest that extensive usage of the film indirectly causes changes in the chemical structure of the enzyme (chemical degradation or precipitation) as a result of the progression of dehydration of the polysaccharide-based film. This is undoubtedly reflected in the alteration of the structure of the polysaccharide-based matrix. 

### 2.3. Morphological Studies of the Biocatalyst via Optical Microscopy

**Figure 4:**
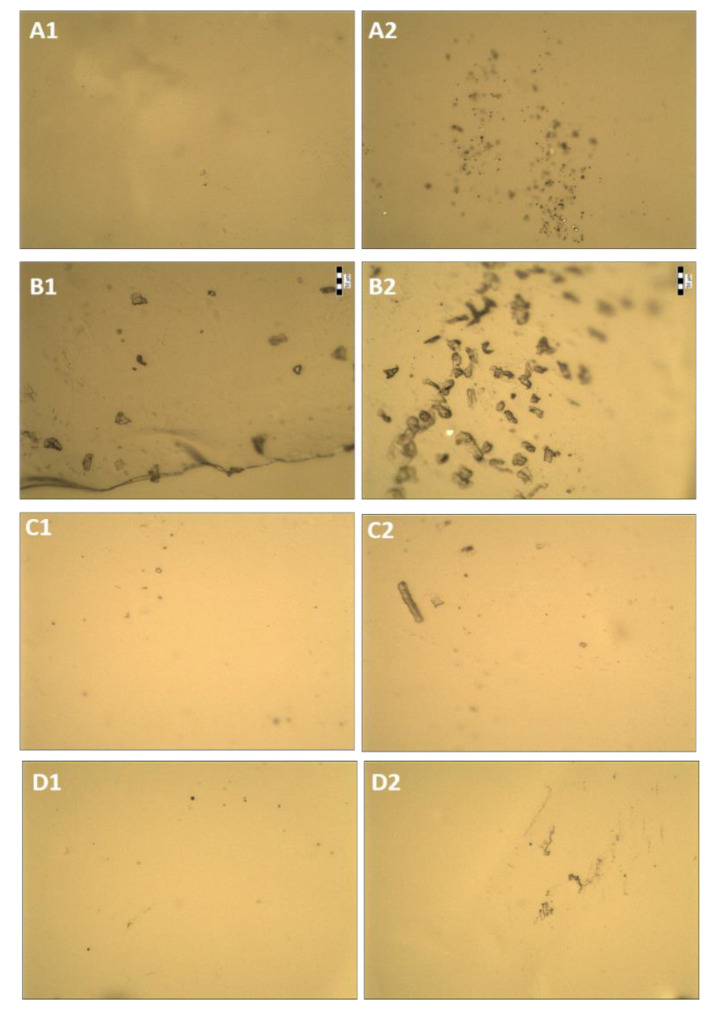
Optical images of unloaded films: (**A1**) and (**A2**) different surface sections of CS film; (**B1**) and (**B2**) different surface sections of HPMC film; (**C1**) and (**C2**) different surface sections of HPMC:CS = 1:1 film; (**D1**) and (**D2**) different surface sections of HPMC:CS = 2:1 film. The scale bar represents 50 μm.

**Figure 5:**
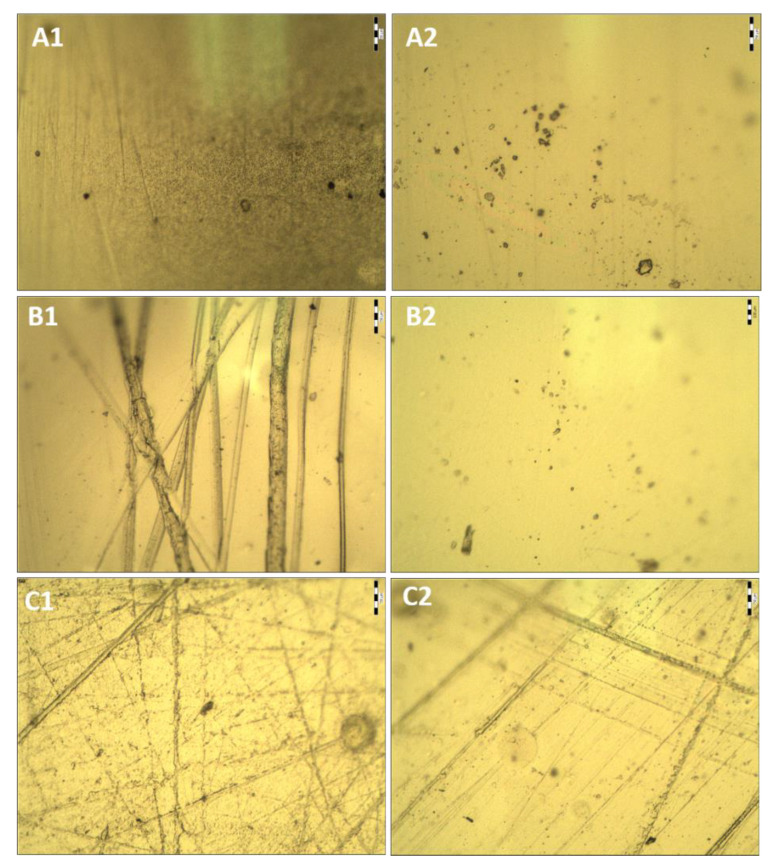
Optical images of HPMC–CS (2:1) unloaded film (**A1**); different surface section of unloaded film (**A2**); enzyme–loaded film (**B1**); different section of enzyme-loaded film (**B2**); enzyme-loaded film after 35 usage cycles (**C1**); different surface section of enzyme-loaded film after 35 usage cycles (**C2**). The scale bar represents 50 μm.

Initial morphological studies showed that the HPMC, CS, and unloaded HPMC–CS films were transparent (Figure 4 and Figure 5). Lipase loading reduced the transparency of the film, as it became slightly opaque with areas of white color and turbidity affecting light transmission. This is probably an effect of crystallization of lipase and/or buffer salts in the film after drying. 

The composition of the matrix, as well as immobilization of the enzyme, clearly influences the appearance of the film (Figure 4). The HPMC film is rather patchy, with numerous bumps and many crystals (approximately 20 μm in diameter), both on the entire surface and inside the film. The CS film is rather smooth, mostly homogeneous, with only a few lumps. It was observed that the higher the film’s CS content, the smoother its surface became (Figure 4B–D). This is probably a result of lower viscosity of the HPMC/CS mixture than that of the HPMC solution. In addition, the film consisting of HPMC and CS is less porous. The polyelectrolyte complexes formed between CS and HPMC cause a change in the degree of dispersion of polymer chains and phase structure, and a change in intermolecular interactions at the interface of the film. Furthermore, it was observed that when the enzyme is incorporated into the film, fewer crystals are visible. A similar result was observed via AFM in our previous study, where the enzyme seemed to level the roughness of the film [21]. To add to this, long, narrow channels appear across most of the surface of the film (Figure 5). This may suggest that immobilization of lipase affects the continuity and mechanical properties of the film’s surface. The film, after repeated use in organic media, seems fatigued, as it has even more longitude marks. The resulting electrostatic complexes are not very stable; therefore, the use of the film under the conditions of an organic solvent may cause structural changes in the film, especially since the biopolymers used are not compatible with isooctane (which was used as a medium for the catalytic reaction).

### 2.4. Microstructural Studies of the Biocatalyst via Scanning Electron Microscopy

**Figure 6:**
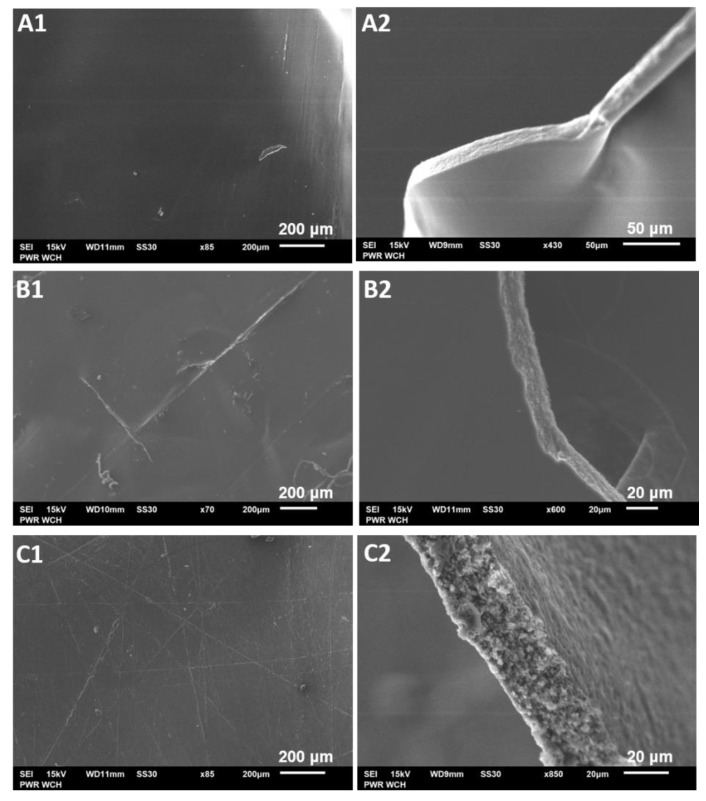
SEM images of (**A1**) unloaded film; (**A2**) cross section of unloaded film; (**B1**) enzyme-loaded film; (**B2**) cross section of enzyme-loaded film (**C1**) enzyme-loaded film after 35 usage cycles. HPMC:CS = 2:1; (**C2**) cross section of enzyme-loaded film after 35 usage cycles.

Scanning electron microscopy (SEM) was used to investigate valuable details of the microstructural changes in the morphology of films in the absence and presence of the enzyme, as well as the effect of repeatedly using the biocatalyst 35 times in organic solvent. SEM images show that the structure of the films differs along with its composition (Figure 6). The surface of all unloaded films, regardless of their composition, was rather smooth, continuous, and even. When the enzyme was loaded, some fine lines appeared. Furthermore, on the surface of the film used, some slight clumps and irregularities occurred (Figure 6C). It is most likely that the lines on the surface of lipase-loaded films were caused by the higher force needed to detach the films from the glass plates, as enzyme addition causes slightly greater stiffness to the film [28].

The cross sections of the films revealed that immobilization of the enzyme slightly changes the structure of the film (Figure 6B2). The film, prior to enzyme loading, due to the structural compatibility between CS and HPMC, was slightly elastic, smooth, compact, and without phase separation. After loading the enzyme, the film became brittle and uneven (lumpy), with a tendency to fracture, but was still nonporous. This indicated that the enzyme was evenly distributed, which is in accordance with results reported in other studies [22,29,30,31]. The film, after repeated use in organic media, became softer and more susceptible to mechanical stress, while multiple shallow pores and granular structures seem to appear inside the enzyme-loaded film after 35 usage cycles. This is clearly visible in the intersection pictures and is probably caused by dehydration of the film, since, when water evaporated during multiple usage cycles, polymer chains and lipase were locally accumulated in the form of granules.

### 2.5. Mechanical and Topographical Characterization

**Figure 7:**
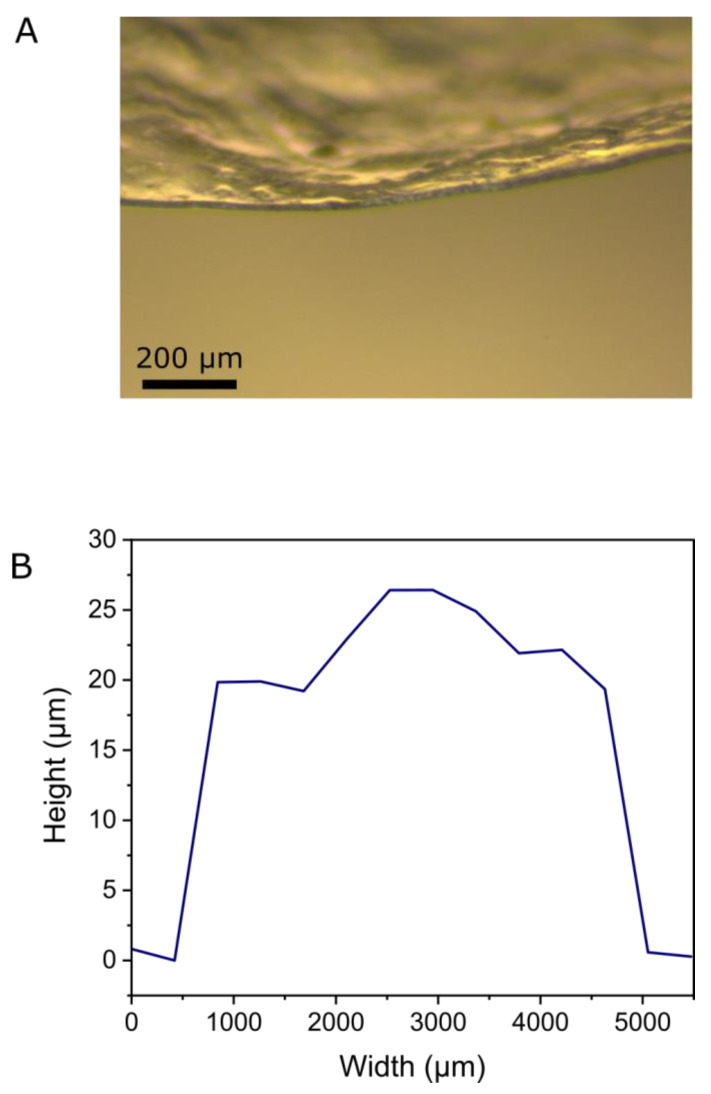
(**A**) Optical micrograph of the edge of an HPMC–CS film. (**B**) Height profile of a section of HPMC–CS film.

To clarify these points, strength analysis took place via mechanical and topographical characterization. The thickness of HPMC–CS film was measured by optical microscopy and profilometry measurements, shown in Figure 7. Optical micrographs of the edge of a film revealed a mean thickness of approximately 16 µm. Profilometry measurements yielded a mean height of 22 µm; however, these measurements also revealed notable fluctuations in height from 19–26 µm across a section of film investigated. Both values appear to be in good agreement with the SEM analysis presented in Section 2.4.

**Figure 8:**
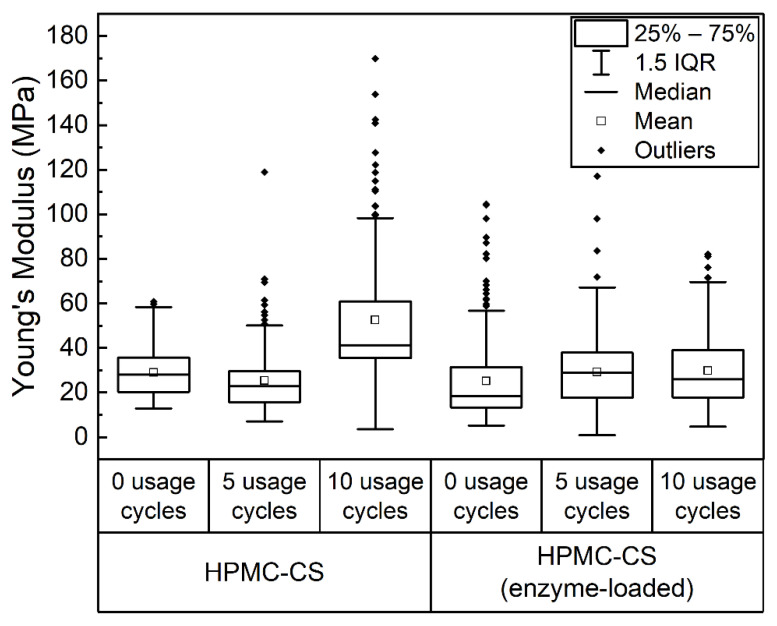
Box plot displaying Young’s modulus values obtained from HPMC–CS films (with and without enzyme loading) before usages, and after 5 and 10 usage cycles. Each plot indicates the distribution of 225 datapoints collected from measurements across a 3 × 3 mm area of film.

The Young’s moduli of HPMC–CS films and their spatial relationships were investigated using microindentation measurements. In this method, a microscale (50 µm diameter) spherical probe was indented into films (folded threefold to ensure minimal influence of the underlying substrate), and force was measured as a function of probe displacement. All materials displayed rate-independent hysteresis between loading and unloading data, typical of an elastoplastic material response, and are therefore best modeled by the Oliver–Pharr approach [32]. In this analysis method, only unloading data are fit (as described in the Methods section), based on the assumption that both plastic and elastic deformation occur during the loading phase, whereas only an elastic response is present during unloading [33]. Arrays of indentation measurements (15 × 15 locations) across a 3 × 3 mm area were made for HPMC–CS and HPMC–CS enzyme-loaded films, prior to use, and after 5 and 10 usage cycles. Young’s moduli maps obtained from these individual tests (along with accompanying microscopy images) are presented in Figures S2 and S3, and are summarized in Figure 8.

The distributions of mechanical properties in Figure 8 are notably broad, with in-dividual measurements (Figures S2 and S3 revealing a high degree of spatial heterogeneity in the stiffness of all films with localized regions of substantially higher Young’s moduli. These broadly distributed mechanical properties could be due to inhomogeneity in film composition from the drying process, or simply a result of the aforementioned variation in film thickness. Importantly, however, no significant differences in the mechanical properties are evident between the two film types, with values of 29.9 ± 10.6 MPa (mean ± SD) and 25.2 ± 19.0 MPa for new films without and with enzyme loading, respectively. Furthermore, statistical analysis showed no substantial changes in the mechanical properties of both types of films after usage, which maintained mean Young’s modulus values between 25 and 30 MPa. The only exception was the HPMC–CS film tested after 10 usage cycles, which, due to the high inhomogeneity of the sample, displayed a much wider distribution of Young’s modulus values and, consequently, a statistically higher mean Young’s modulus of 52.5 ± 26.7 MPa. However, in general, the stability of the Young’s modulus of these films is in good agreement with previous findings that films could undergo up to 35 usage cycles [21].

## 3. Conclusions

Hydroxypropyl)methyl cellulose and chitosan were combined to form a film which was used to immobilize lipase from *Mucor miehei*. The main aim of the present work was to structurally investigate the empty and enzyme-loaded film. Enzyme loading onto the matrix appears to have very little effect on the structure of the film. Mixing the original HPMC and CS solutions resulted in an increase of the solution’s pH, and adding the enzyme resulted in an even more neutral surface charge. The presence of a combination of –NH_2_ in plane bending and C–N stretching vibrations showed the formation of intermolecular hydrogen bonds between the amide groups of two polymers with the lipase. Weak electrostatic interactions between the polysaccharides and the lipase were observed. Regarding the reuse of the biocatalyst, it can be stated that the texture became quite fatigued, and in the intersection some granules could be observed. This could be due to the moisture reduction of the matrix after repeated use. Furthermore, no significant differences in the mechanical properties were evident between the loaded and unloaded films. The two polymers seem to have good interaction, and taking into account the drying and loading with enzyme process, which does not undergo any smoothing steps, the film offers a stable environment with enough water for the enzyme to perform multiple times.

## 4. Materials and Methods

### 4.1. Materials

The films prepared within this study were based on two natural polymers (hydroxypropyl)methyl cellulose (HPMC) (3600–5500 mPa.s), which was obtained from Sigma, Darmstadt, Germany, and chitosan (CS) from shrimp and other crustacean shells (viscosity 200–600 mPa.s, 0.5% in 0.5% acetic acid, 20 °C; deacetylation value: 80%, molecular mass 1526.464 g/mol), which was purchased from TCI, Belgium. Lauric acid was obtained from Sigma, Darmstadt, Germany. Acetic acid was obtained from LachNer, Neratovice, Czech Republic. All other materials were at least reagent grade. Millipore Milli-Q water was used for the preparation of gels and buffer solutions. Lipase from *Mucor miehei* (*M. miehei*) was supplied by Fluka, Basel, Switzerland, and had a specific activity of 1.19 U/mg of protein (1 U corresponds to the amount of enzyme which liberates 1 μmol butyric acid per min at pH 8.0 and 40 °C using tributyrin as substrate). 

### 4.2. Methods

#### 4.2.1. Film Preparation

Films based on HPMC–CS were prepared by diluting HPMC in distilled water and CS in 1% acetic acid, resulting in a solution of 2% *w*/*w*, each. The two solutions were left overnight until fully transparent and homogeneous. Then, a mixture of them was prepared in the right ratio, for each experiment, and placed on a Petri dish to dry overnight. In the case of single-polymer film, only one of the two polymers’ solutions was placed on a Petri dish. After drying, approximately 0.3 g of films were produced. After the two solutions were mixed, no phase separation could be observed regardless of the composition, showing the fine biopolymers’ compatibility. The procedure is illustrated in Supplementary Materials Figure S1.

#### 4.2.2. Enzyme-Loaded Films Preparation

To prepare the biocatalyst (HPMC–CS film loaded with lipase (2:1)), the appropriate amount of Tris/HCl buffer, pH 7.5 containing lipase, was added to the polymers’ solution, prior to drying. In a typical experiment, 1 g of CS solution 2% *w*/*w* was mixed with 2 g of HPMC solution 2% *w*/*w*, followed by the addition of 30 μL of buffer containing 0.3 mg lipase and stirred gently at room temperature. The final mixture was left overnight on a Petri dish to dry, thus formulating a film in which the enzyme was immobilized. After peeling the film from the Petri dish, it was washed three times with 5 mL of isooctane to remove any enzyme molecules that may not be effectively immobilized on the network and leak to the reaction solvent. The final biocatalyst produced contained 1 mg lipase/g of film.

#### 4.2.3. Reuse of Film

In the case of reused films, the biocatalyst (HPMC:CS film loaded with lipase (2:1)) was used for the repeated catalysis of propyl laurate synthesis. For this purpose, approximately 0.3 g of a film containing 1 mg lipase/g of film was placed in a screw-cap bottle with 10 mL of the appropriate organic solvent containing lauric acid and 1-propanol (100 mM each). Each day, the film was placed in a new bottle with fresh solvent and substrates, and after several uses, part of the film was cut and studied.

#### 4.2.4. Small-Angle X-ray Scattering (SAXS) Measurements

SAXS experiments for films in the presence and absence of the immobilized enzyme or in the presence or absence of glycerol were performed on an XEUSS 2.0 (XENOCS, Grenoble, France) with a GeniX 3D source delivering a 8 keV beam coupled to an FOX 3D single reflection optical mirror centered on the Cu Kα radiation (λ = 1.54 Å). The beam was further collimated and defined by a set of two motorized scatterless slits. The samples were placed in capillaries for solid samples and were folded twice to obtain a stronger signal. The samples, under vacuum as the whole flight path from the FOX3D mirror to the detector window, were exposed for 3 hours. The data were collected by a two-dimensional PILATUS-300k detector (DECTRIS, Baden-Dättwill, Switzerland) placed perpendicular to the direct beam at a distance of 1634 mm, calibrated with a Silver behenate standard. Rectangular images with shape (487, 619—horizontal, vertical) were obtained and further processed with the FOXTROT 3.4.9-3471 software (collaboration between XENOCS (Grenoble, FRANCE) and the SOLEIL synchrotron (Gif-sur-Yvette, FRANCE) SWING beamline team), giving access to a range of scattering wave vectors q from typically 0.007 Å^−1^ to 0.24 Å^−1^.

#### 4.2.5. Fourier Transform Infrared Spectroscopy (FTIR)

Fourier transform infrared (FTIR) spectroscopy was used to investigate the functional groups of the polymers that made up the film to determine the possible interactions between their functional groups and the enzyme in the composite systems. The samples were analyzed between 4000 to 400 cm^−1^ with a resolution of 2 cm^−1^ using a Bruker VERTEX 70 V vacuum spectrometer (Bruker Optik GmbH, Birrika, MA, USA) equipped with a diamond attenuated total reflectance (ATR) accessory and Opus software (Bruker Optik GmbH, Ettlingen, Germany) for the spectra analysis.

#### 4.2.6. Microscopic Studies

The film morphology and microstructure were examined via optical microscopy and scanning electron microscopy (SEM). Newly prepared and 35-times repeatedly used enzyme-loaded films, as well as unloaded films, were deposited on a glass slide and subjected to microscopic observations via an optical microscope 41-CX (Olympus, Japan) (with 10× magnification) equipped with a 500MI digital camera (Ataray, Turkey). Quick-photo 2.2 software was used to capture and analyze pictures. Films were also attached to a double-sided carbon tape, sputtered with carbon, and examined using a JSM-6601LV scanning electron microscope (JEOL, Akishima, Japan) (operating voltage 15 kV). 

#### 4.2.7. Physiochemical Studies

The moisture content of the HPMC–CS films was determined gravimetrically using the moisture analyzer MB27 (Ohaus, Nänikon, Switzerland), and the moisture content was estimated as a percentage of weight loss after 30 min at 100 °C. The experiment was performed three times for each type of film studied. The electrophoretic zeta potential (ζ) of polyelectrolyte solutions was measured with a Zetasizer Nano ZS (ZEN3600) from Malvern Instruments (UK) at 25 °C equipped with a HeNe laser (632.8 nm) and using a noninvasive backscatter (NIBS) technology. For these measurements, the following solutions were prepared: 2% CS, 2% HPMC, HPMC–CS 2:1 *w*/*w*, and HPMC–CS 2:1 *w*/*w* with enzyme. The measurements were performed thrice for each solution.

#### 4.2.8. Mechanical and Topographical Studies

The mechanical properties and topography of the HPMC–CS films were determined using an FT-MTA03 (FemtoTools AG, Buchs, Switzerland) equipped with an FT-S2000 microforce sensing probe (range: ±2000 µN, resolution: 0.005 µN) capped with a 50 µm borosilicate glass sphere (BSGMS-2.2 from Cospheric).

For profilometry tests, a single layer of film (approximately 0.5 cm wide) was held taut over a glass slide. Indentation measurements were made from a fixed height across the width of the film segment and surrounding glass slide. A stick-slip actuator (29 mm vertical range, 1 nm positional resolution) was used, operating in a stepped mode (with 0.5 µm steps and a 0.02 s delay), at a speed of 50 µm/s, until a force of 1500 µN was reached. Data were analyzed by determining the vertical position at which a force of 1000 µN was reached, and calculating the corresponding material height compared to the glass slide.

For mechanical tests, films were first folded into a 3-layer structure, then compressed between two glass slides prior to measurement. Samples were then placed on a glass slide, whilst a light pressure was applied to the edges of the sample with additional glass slides to maintain sample flatness throughout measurement. Measurements were conducted using a piezoscanner (50 µm vertical range, 0.1 nm positional resolution) in a continuous actuation mode to indent the probe into the sample. The film surface was found by applying a force threshold of 3 µN, then retracting the probe 1 µm from the surface to allow the acquisition of baseline data. Measurements were then carried out at a speed of 1 µm s^−1^, to a maximum force of 30 µN (corresponding to a maximum indentation depth of approximately 0.5 µm), with data collected at a frequency of 200 Hz. Data were collected throughout the approach and indentation into the material (loading) and retraction back to the initial probe position (unloading).

Data were analyzed using a custom Python-based application. For each set of force–displacement data, a contact point was firstly determined by the location of a maximum in the second derivative of force as a function of displacement. Unloading data beyond the contact point were then analyzed using the method described by Oliver and Pharr outlined below [33,34]. A total of 50% of the data with greatest indentation depth were fitted to a power law of the form:

(1) $$P=\alpha {\left(h-h_{f}\right)}^{m}$$

where *P* is the load at displacement *h*, *m* is an exponent set to 1.5 for a spherical indentation probe, and α and $h_{f}$ are fitting parameters. From these parameters, stiffness (S=dP/dh) at peak load ($P_{max}$) is determined. ${SP}_{max}$ are then used to calculate the contact depth ($h_{c}$) and contact area ($A_{c}$) using:

(2) $$h_{c}=h_{max}-0.75\times \frac{P_{max}}{S}$$

(3) $$A_{c}=\pi \left(2Rh_{c}-h_{c}{}^{2}\right)$$

where $h_{max}$ is the maximum displacement. The reduced modulus ($E_{R}$) is then given by:

(4) $$E_{R}=\frac{S\sqrt{\pi }}{S\sqrt{A_{c}}}$$

Finally, the Young’s modulus of the sample ($E_{S}$) is given by:

(5) $$\frac{1}{E_{R}}=\frac{1-\nu_{S}{}^{2}}{E_{S}}+\frac{1-\nu_{P}{}^{2}}{E_{P}}$$

where $E_{P}$ and $\nu_{P}$ are the Young’s modulus and Poisson’s ratio of the probe (values for borosilicate glass of 63 Gpa and 0.2 were used), and $\nu_{S}$ is the sample Poisson’s ratio, for which a value of 0.4 was used and is typical for biopolymer materials [35].

## Data Availability

SAXS data available upon request at Centre de recherche Paul-Pascal, Pessac, France.

## Supplementary Materials

The following supporting information can be downloaded at: https://www.mdpi.com/article/10.3390/gels8090595/s1, Figure S1: Step-by-step procedure for the formation of the loaded film with enzyme. (A) Powders of the two polymers, HPMC and chitosan; (B) dilution of the two polymers in distilled water and acidic water (1%) for HPMC and chitosan, respectively; (C) overnight solution of the two polymers; (D) mix of the two solutions on the Petri dish (2:1 ratio HPMC:chitosan); (E) addition of the enzyme buffer; (F) gentle stirring with spatula to homogenize the liquid; (G) after overnight drying, we peeled off the dried film from the Petri dish; Figure S2: Mechanical characterization of HPMC–CS films: Optical micrographs of the films used for mechanical testing before usage (A), after 5 usage cycles (C), and after 10 usage cycles (E), with testing area and coordinates shown. Young’s modulus maps from areas depicted in optical micrographs for films before usage (B), after 5 usage cycles (D), and after 10 usage cycles (F). Colormap maintained for comparison between samples; Figure S3: Mechanical characterization of enzyme-loaded HPMC–CS films: Optical micrographs of the films used for mechanical testing before usage (A), after 5 usage cycles (C), and after 10 usage cycles (E), with testing area and coordinates shown. Young’s modulus maps from areas depicted in optical micrographs for films before usage (B), after 5 usage cycles (D), and after 10 usage cycles (F). Colormap maintained for comparison between samples.
