# Supplementary material for: (Hydroxypropyl)methyl Cellulose-Chitosan Film as a Matrix for Lipase Immobilization—Part ΙΙ: Structural Studies
Source: Gels. 2022 Sep 17;8(9):595. doi: 10.3390/gels8090595 (PMC9498736; doi:10.3390/gels8090595)
Supplement: Supplementary file 1 [file gels-08-00595-s001.zip › gels-1891043-supplementary.pdf]

gels

MDPI

Article

(Hydroxypropyl)methyl Cellulose-Chitosan Film as a Matrix for Lipase Immobilization. Part II: Structural Studies

Evdokia Vassiliadi [1,2], Marta Tsirigotis-Maniecka [3], Henry E. Symons [4], Pierangelo Gobbo [5], Frédéric Nallet [6], Aristotelis Xenakis [1] and Maria Zoumpanioti [1,*]

Supplementary material

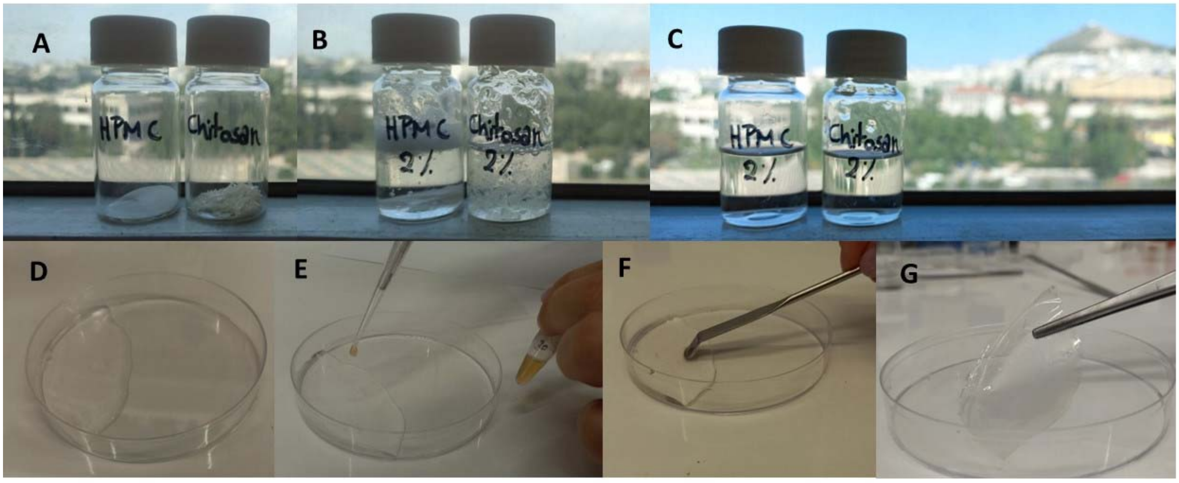

Figure S1. Step by step procedure for the production of the enzyme-loaded film. (**A**) The two polymers, HPMC and Chitosan; (**B**) Dilution of the two polymers in distilled water and acidic water (1%) for HPMC and Chitosan, respectively. They are then left overnight; (**C**) solutions of the two polymers, the next day; (**D**) Mix of the two polymer solutions on a petri dish, HPMC:Chitosan=2:1; (**E**) Addition of the enzyme-containing buffer; (**F**) Gentle stirring with spatula to homogenize the liquid; (**G**) After overnight drying, the film can be pilled off of the petri dish.

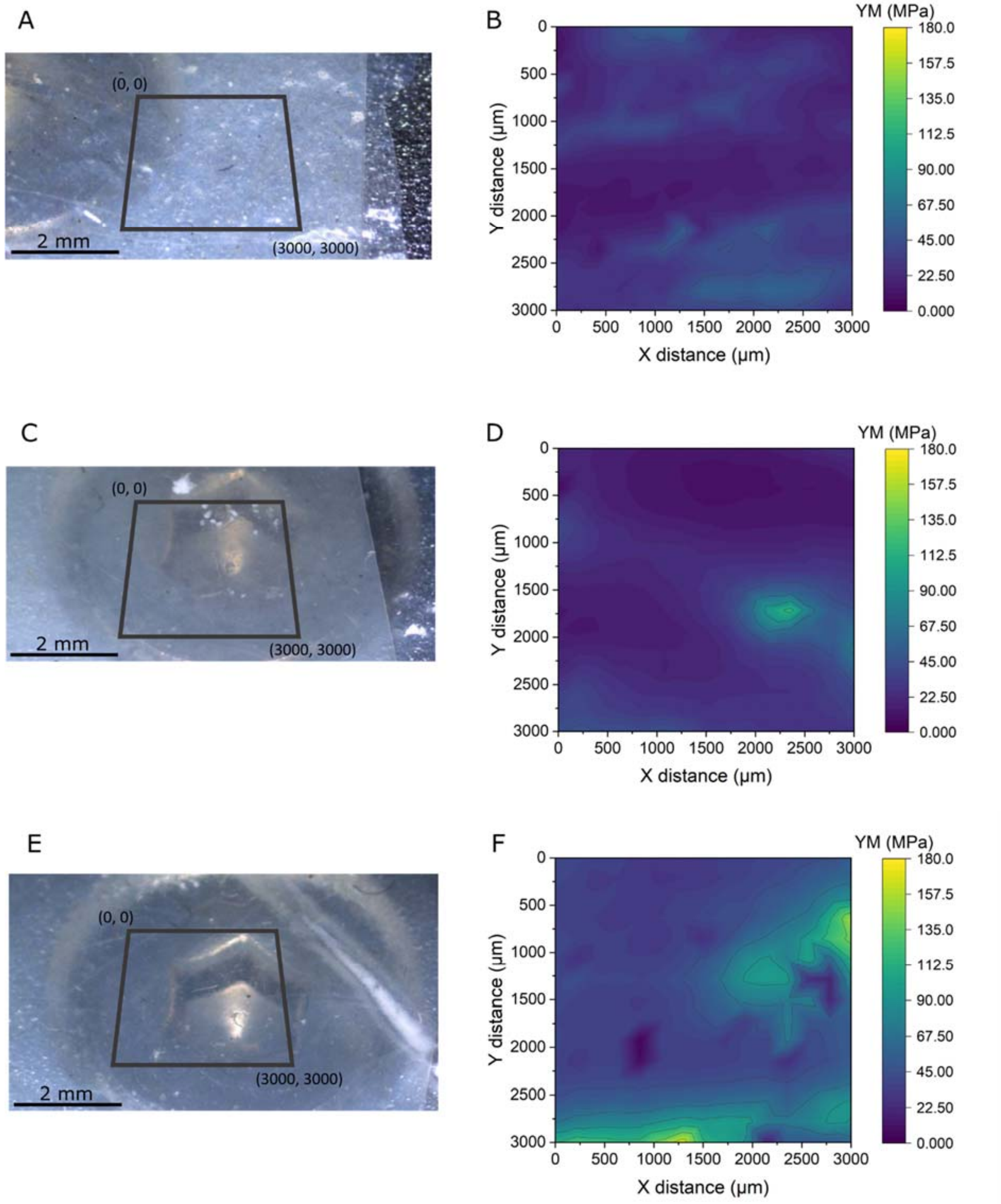

Figure S2. Mechanical characterization of HPMC-CS films: Optical micrographs of the films used for mechanical testing before usage (**A**), after 5 usage cycles (**C**), and after 10 usage cycles (**E**), with testing area and coordinates shown. Young's modulus maps from areas depicted in optical micrographs for films before usage (**B**), after 5 usage cycles (**D**), and after 10 usage cycles (**F**). Colormap maintained for comparison between samples.

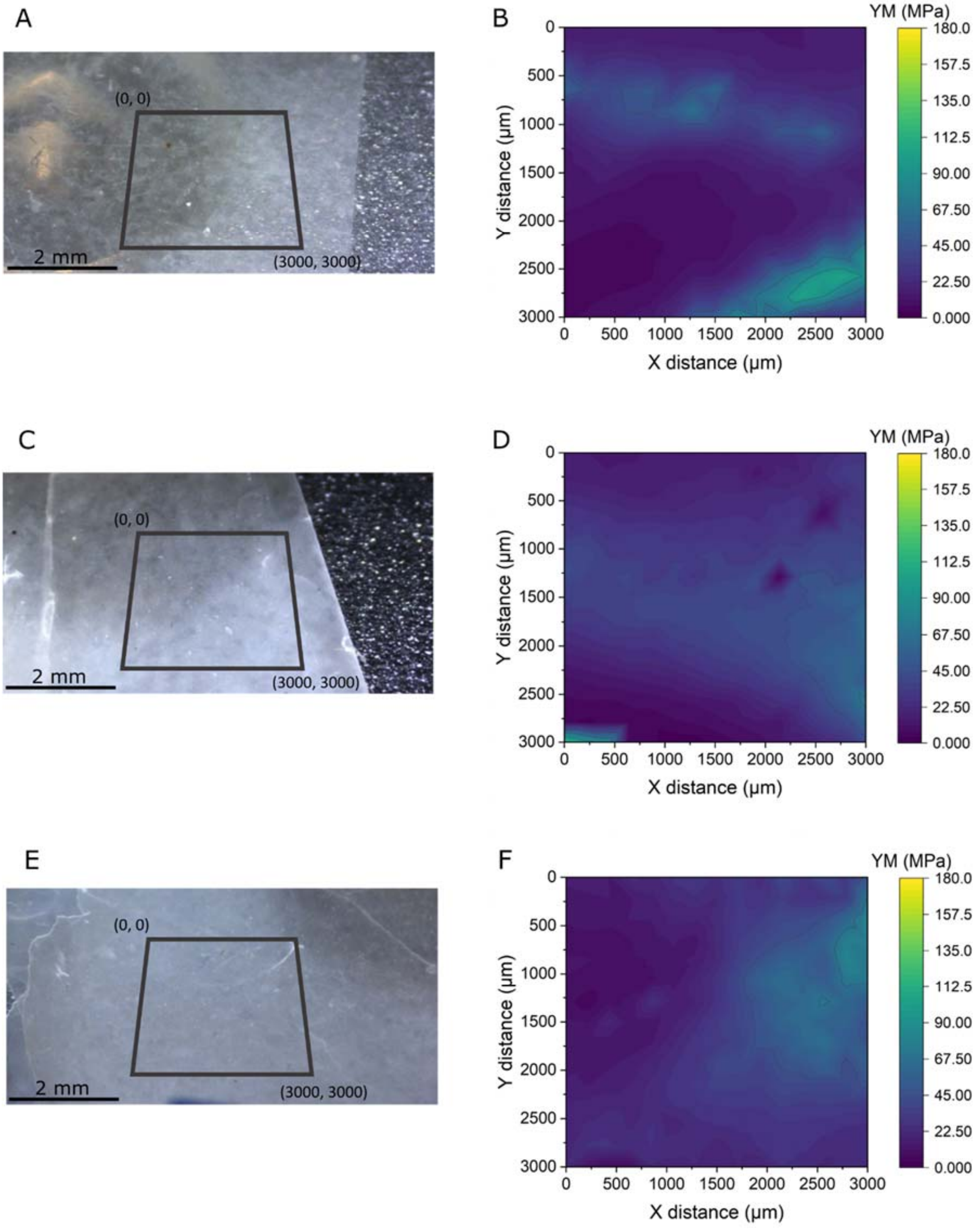

Figure S3. Mechanical characterization of enzyme-loaded HPMC-CS films: Optical micrographs of the films used for mechanical testing before usage (**A**), after 5 usage cycles (**C**), and after 10 usage cycles (**E**), with testing area and coordinates shown. Young's modulus maps from areas depicted in optical micrographs for films before usage (**B**), after 5 usage cycles (**D**), and after 10 usage cycles (**F**). Colormap maintained for comparison between samples.
